# A Standardized Aroma Lexicon and Sensory Wheel for Characterizing Aroma Quality of Traditional Korean *Doenjang*

**DOI:** 10.1111/1750-3841.71481

**Published:** 2026-09-13

**Authors:** Ji‐Sun Hwang, In‐Seo Hwang, Mina K. Kim

**Affiliations:** ^1^ Department of Food Science and Human Nutrition and K‐Food Research Center Jeonbuk National University Jeonju‐si Jeonbuk Republic of Korea

**Keywords:** aroma diversity, aroma lexicon, aroma wheel, descriptive sensory analysis, fermented soybean paste, traditional *doenjang*

## Abstract

Traditional Korean *doenjang* is a spontaneously fermented soybean paste with complex and variable aroma characteristics, but a standardized sensory language for describing its aroma quality has not been established. This study developed a reference‐based aroma lexicon and aroma wheel for traditional Korean *doenjang* and evaluated their applicability for sensory characterization. Eighty‐one traditional *doenjang* samples collected from nine regions of Korea were evaluated by a trained descriptive sensory panel. The panel identified 80 aroma descriptors, which were defined with reference standards and organized into 10 first‐level and 19 second‐level categories, including fermented soybean‐related, fish‐related, fruity, sweet, dairy‐associated, lipid‐related, vegetable‐like, roasted, grain‐like, spicy, sour, and miscellaneous aroma characteristics. All descriptors significantly discriminated among samples after false discovery rate correction (*q* < 0.05), and the median eta‐squared and omega‐squared values were 0.885 and 0.878, respectively, indicating strong sample‐related variation. Hierarchical cluster analysis supported the organization of major descriptor groups, particularly fermented soybean‐related, fruity, dairy‐associated, roasted, vegetable‐associated, and fish‐related attributes. Principal component analysis showed broad dispersion of the samples, suggesting that aroma profiles were influenced more by differences among individual traditional *doenjang* products than by geographic origin alone. An applicability assessment using 12 representative samples selected from the original sample set supported the practical coverage of the established lexicon without requiring additional major descriptors. These results provide a standardized sensory framework for describing the aroma quality of traditional Korean *doenjang* and support its use in quality evaluation, product development, sensory communication, and future sensory–instrumental studies of fermented soybean foods.

**Practical Applications:**

The aroma lexicon and sensory wheel developed in this study can help producers, researchers, and quality control personnel describe the aroma quality of traditional Korean *doenjang* using consistent terms. The full lexicon provides a comprehensive reference framework, while selected descriptors from major aroma categories can be used as a reduced working lexicon for product‐specific quality evaluation, sensory training, product development, and communication of aroma differences among fermented soybean products.

## 1 Introduction

Traditional *doenjang*, also known as *hansik doenjang*, is a Korean fermented soybean paste that has long been used as a major seasoning ingredient in Korean cuisine. Unlike commercially manufactured *doenjang*, traditional *doenjang* is generally produced using soybeans, salt, and water without the addition of defined starter cultures, flavor enhancers, or other additives (Shin et al. 2023). The production process includes the preparation of *meju*, a naturally fermented soybean block, followed by brining and long‐term aging under variable environmental conditions (Hong and Kim 2020; Park et al. 2002). Because traditional *doenjang* fermentation largely depends on naturally occurring microorganisms and non‐standardized fermentation environments, considerable variation can occur in microbial succession, metabolite production, and aroma formation during fermentation (Chun et al. 2020; Han et al. 2021).

Aroma is one of the most important quality attributes of traditional *doenjang* because it directly affects product identity, sensory perception, and consumer acceptance. The aroma characteristics of traditional *doenjang* are highly complex and are influenced by multiple factors, including raw materials, salt concentration, fermentation temperature, aging period, regional climate, and microbial ecology (Han et al. 2021; Kim et al. 2016; Song et al. 2021). Previous studies have shown that fermentation conditions can affect volatile compound formation and sensory characteristics in soybean‐fermented foods such as *doenjang*, *ganjang*, *gochujang*, and *miso* (Devanthi and Gkatzionis 2019; Han et al. 2020; Kumazawa et al. 2013; Ramalingam et al. 2022). Traditional *doenjang* has been reported to show diverse aroma characteristics, including fermented soybean, roasted, fruity, dairy, fishy, sour, and earthy notes, which are generated through complex biochemical and microbial metabolism and biochemical reactions, including proteolysis, lipolysis, and Maillard reactions, during spontaneous fermentation (Baek 2017; Hong et al. 2012; Jang et al. 2021). This aroma complexity is an important sensory feature of traditional *doenjang*, but it also makes consistent quality evaluation and sensory communication difficult.

Descriptive sensory analysis is a useful method for characterizing complex food products using trained panelists, standardized terminology, and reference‐based intensity evaluation (Kemp et al. 2018; Lawless and Heymann 2010). Sensory lexicons and sensory wheels have been developed for various foods and beverages, including coffee, chocolate, tea, olive oil, marine oils, and pulse‐derived ingredients, to organize complex sensory information into structured and reproducible frameworks (Chambers et al. 2016; Chigwedere et al. 2022; De Pelsmaeker et al. 2019; Koch et al. 2012; Larssen et al. 2018; Mojet and de Jong 1994). A well‐developed sensory lexicon provides clearly defined descriptors and reference standards, which can improve consistency in sensory evaluation and communication among researchers, industry professionals, and product developers (Lawless and Civille 2013; Murray et al. 2001). In addition, sensory wheels can serve as practical visualization tools for sensory training, product comparison, quality control, and the interpretation of sensory differences across products.

Several studies have investigated the sensory characteristics of *doenjang* using descriptive sensory analysis (Jang et al. 2021; Jung et al. 2017; Kim et al. 2010; Shin et al. 2022; Shin et al. 2023). These studies reported aroma descriptors related to fermented soybean products, including *meju*, soy sauce, fish sauce, alcohol, cheese, fruit, roasted, and earthy characteristics. However, most previous studies focused on specific sample sets, consumer preference, fermentation conditions, or processing effects, and the descriptors used across studies were not fully standardized. Therefore, it remains difficult to compare sensory results across studies or to apply a consistent aroma language for quality evaluation of traditional *doenjang*. To date, no comprehensive reference‐based aroma lexicon or aroma wheel has been developed to systematically describe the aroma quality of traditional Korean *doenjang* using a broad and diverse sample set.

Therefore, the objective of this study was to develop a standardized, reference‐based aroma lexicon and aroma wheel for traditional Korean *doenjang* and to assess their applicability for sensory characterization. A large set of traditional *doenjang* samples collected from different regions of Korea was evaluated by trained descriptive sensory analysis. The resulting sensory framework was intended to support consistent aroma description, sensory quality evaluation, product development, and future sensory–instrumental studies of fermented soybean foods.

## 2 Materials and Methods

### 2.1 Sample Preparation

Traditional Korean *doenjang* samples were selected to represent a broad range of products differing in regional origin, ingredient composition, and manufacturing practices. A total of 81 traditional *doenjang* samples were obtained from nine regions of Korea: Gyeonggi (GG, *n =* 6), Gangwon (GW, *n =* 6), Chungnam (CN, *n =* 13), Chungbuk (CB, *n =* 15), Jeonnam (JN, *n =* 10), Jeonbuk (JB, *n =* 14), Gyeongnam (GN, *n =* 5), Gyeongbuk (GB, *n =* 7), and Jeju (JJ, *n =* 5). Detailed sample information is provided in Table S1, and the geographical distribution of the samples is shown in Figure S1.

The samples were purchased from local producers through regional online markets to reflect the diversity of traditionally manufactured *doenjang* products available in Korea. As shown in Table S1, the samples varied in ingredient composition, including soybean and *meju* ratios, salt concentration, use of refined water, and incorporation of region‐specific ingredients. Because traditional *doenjang* is produced by spontaneous fermentation under variable manufacturing and environmental conditions, this sample set was considered suitable for developing a broad aroma lexicon and sensory wheel for traditional *doenjang*. All samples were stored at 4°C until analysis. For sensory evaluation, 5 g of each sample was placed in a 56‐mL opaque plastic cup with a lid and labeled with a randomized three‐digit code. The prepared samples were equilibrated at room temperature at 25°C for 2 h before evaluation. To minimize sensory fatigue and maintain panel consistency, 8 to 10 samples were evaluated per session.

### 2.2 Descriptive Analysis

#### 2.2.1 Panel Training

The sensory panel consisted of seven highly trained panelists, including six females and one male aged 20 to 42 years, from the Sensory Science and Flavor Chemistry Laboratory at Jeonbuk National University, Jeonju, Republic of Korea. All panelists had more than 200 h of prior experience in descriptive sensory analysis using the Spectrum method and the 15‐point universal intensity scale (Lawless and Civille 2013; Meilgaard et al. 1999). The lead panelist had more than 1,000 h of experience evaluating various food products, particularly fermented soybean products, using the same methodology.

Before formal evaluation, panelists completed approximately 30 h of additional training focused on aroma recognition, intensity calibration, use of reference standards, and development of aroma descriptors for traditional *doenjang*. During training, panelists were repeatedly exposed to candidate reference standards and practiced assigning intensity ratings using the 15‐point universal intensity scale. Descriptor definitions, reference materials, and reference intensity values were refined through consensus discussion until the panel reached agreement on descriptor usage and intensity anchoring. Repeated practice evaluations were conducted before formal data collection to improve consistency in aroma recognition and intensity scaling.

#### 2.2.2 Aroma Sensory Lexicon Development

Before each evaluation session, panelists were instructed to avoid smoking, alcohol consumption, perfume use, and intake of strongly flavored foods and beverages, including coffee, to minimize sensory adaptation and maintain aroma sensitivity. The aroma sensory lexicon was developed using an iterative consensus‐based procedure based on descriptive sensory analysis principles described by Lawless and Civille (2013). The procedure included three steps: descriptor generation, refinement and standardization of descriptors with corresponding definitions and references, and aroma intensity evaluation using the 15‐point universal intensity scale.

All 81 traditional *doenjang* samples were evaluated at least once during the descriptor‐generation stage over multiple sensory evaluation sessions. As additional samples were introduced, panelists generated and discussed aroma descriptors through an iterative consensus‐based process. Selected samples were revisited when descriptor definitions, distinctions among related terms, or reference standards required further refinement. Newly generated descriptors were discussed, refined, merged, or eliminated through panel consensus until all 81 samples had been evaluated and no additional relevant aroma descriptors emerged. The generated terms were then reviewed through panel discussion to remove redundant, overlapping, hedonic, or ambiguous expressions. Terms with similar sensory meanings were merged, and descriptors were retained when the panel agreed that they were recognizable, relevant to traditional *doenjang* aroma, and sufficiently distinct from other descriptors. The retained descriptors were standardized with consensus definitions and reference materials. Reference standards and corresponding intensity values were established through repeated panel discussions and used as anchors for panel calibration and intensity scaling. The reference materials were commercially obtained or prepared according to the procedures specified in Table 1. Each reference was placed in a lidded, opaque white plastic cup with a diameter of 70 mm and presented to the panelists for orthonasal evaluation. During training, panelists repeatedly smelled the references while reviewing the corresponding descriptor definitions and reference intensity values on the 15‐point universal intensity scale. The references were used to establish a shared understanding of each aroma descriptor and to anchor intensity ratings across panelists.

**TABLE 1:** Definitions and references of 80 aroma lexicons identified in traditional *doenjang* produced in Korea.

1^st^ category	2^nd^ category	Aroma attributes	Definitions	References	
				References	Intensity
Fermented	Bean‐related	Cheonggukjang	Characteristic aromatics associated with cheonggukjang	Cheonggukjang, wanjeon food, Gwangju, Korea	4
		Chunjang	Characteristic aromatics associated with chunjang	Chunjang powder, Nongshim, Seoul, Korea	3.5
		Meju	Characteristic aromatics associated with meju powder	Fermented soybean powder, Chodam food, Gwangju, Korea	3.5
		Soy sauce	Characteristic aromatics associated with soy sauce	Soy sauce 501, Sempio, Seoul, Korea	4.5
		Steamed bean	Characteristic aromatics associated with cooked soybean	Cooked soybean at 121°C for 15 minutes, Jeonju, Korea	3
		Tofu	Characteristic aromatics associated with fresh tofu	Tofu, Pulmuone, Seoul, Korea	1
		Tsuyu sauce	Characteristic aromatics associated with tsuyu sauce	Tsuyu sauce, Kikkoman food, Japan	3
	Dairy	Cheddar cheese	Characteristic aromatics associated with Cheddar cheese	Cheddar slice cheese, Seoul milk, Seoul, Korea	2.5
		Feta cheese	Characteristic aromatics associated with Feta cheese	Feta cheese, Kolios, Kilkis, Greece	3
		Yogurt	Characteristic aromatics associated with yogurt	Bio plain yogurt, Maeil, Seoul, Korea	2
		Butter	Characteristic aromatics associated with butter	Unsalted butter, Seoul milk, Seoul, Korea	2.5
Fish	Fermented‐fishy	Ammonia	Characteristic aromatics associated with fermented fish	Fully ripen skate fish	5
		Fish sauce	Characteristic aromatics associated with anchovy fish sauce	Anchovy sauce, Chungjungone, Sunchang, Jeollabuk‐do, Korea	4
		Fishy	Characteristic aromatics associated with fresh fish	Fish, E‐mart, Jeonju, Korea	3
		Tuna can	Characteristic aromatics associated with canned tuna	Canned tuna, Dongwon, Seoul, Korea	2
	Dried‐fishy	Dried filefish	Characteristic aromatics associated with dried filefish	Dried filefish, B&B Food, Seongnam, Korea	3
		Dried squid	Characteristic aromatics associated with dried squid	Dried squid, Seongho food, Pohang, Korea	2.5
		Roasted seaweed	Characteristic aromatics associated with roasted seaweed	Roasted seaweed for gimbap, no brand, Seoul, Korea	1
Fruit	Fruit	Apple	Characteristic aromatics associated with freshly cut apple	Fresh apple, Jeonju, Korea	1.5
		Dried cranberry	Characteristic aromatics associated with dried cranberry	Dried cranberry, Dongwoo food, Gwangju, Korea	2
		Dried fig	Characteristic aromatics associated with dried fig	Dried fig, Taylor Farms, CA, USA	1.5
		Dried jujube	Characteristic aromatics associated with dried jujube	Dried jujube, Jeonju, Korea	2
		Grape	Characteristic aromatics associated with grape juice	Dr. Kim's grape juice, Seoul, Korea	2.5
		Pear	Characteristic aromatics associated with pear	Pear, Jeonju, Korea	1.5
		Pineapple	Characteristic aromatics associated with pineapple	Freshly cut pineapple, Dole, CA, USA	2
		Quince	Characteristic aromatics associated with quince	Quince, Jeonju, Korea	1
		Raisin	Characteristic aromatics associated with raisin	non‐pesticide‐treated raisin, Chamdaol, Namyangjoo, Korea	2.5
		Strawberry	Characteristic aromatics associated with strawberry	Strawberry, Jeonju, Korea	2
		Tomato	Characteristic aromatics associated with freshly cut tomato	Tomato, Jeonju, Korea	1.5
		Tropical fruits	Characteristic aromatics associated with canned tropical fruits	Tropical fruit cocktail, Del monte, CA, USA	2.5
Sweet aromatics	Sweet aromatics	Black taffy	Characteristic aromatics associated with black taffy	Black taffy, Dongshin Food, Korea	2
		Brown sugar	Characteristic aromatics associated with brown sugar	Brown sugar, CJ CheilJedang, Korea	1.5
		Coke aroma	Characteristic aromatics associated with coke	Coca‐cola, Coca‐cola, GA, USA	2
		Sweet aromatics	Characteristic aromatics associated with syrup	Sucrose syrup	2.5
		Chocolate	Characteristic aromatics associated with dark chocolate	Dark chocolate, E‐mart, Seoul, Korea	2
	Floral	Candy	Characteristic aromatics associated with candy	Chupachups, Nongshim, Seoul, Korea	1.5
		Honey	Characteristic aromatics associated with honey	Honey, Dongseo, Seoul, Korea	2.5
		Flower	Characteristic aromatics associated with flower	Flower, Jeonju, Korea	2.5
Fatty	Fatty	Oily	Characteristic aromatics associated with oil	Canola oil, CJ Cheiljedang, Seoul, Korea	1
		Rancid	Characteristic aromatics associated with oxidized oil	Oxidized canola oil, CJ Cheiljedang, Seoul, Korea	1.5
		Sesame oil	Characteristic aromatics associated with sesame oil	Sesame oil, Ottugi, Seoul, Korea	2
	Waxy	Crayon	Characteristic aromatics associated with crayon	Crayon, Colorpencil, Seoul, Korea	2
Vegetable	Vegetable	Cucumber	Characteristic aromatics associated with cucumber	Cucumber, Jeonju, Korea	2
		Olive	Characteristic aromatics associated with canned olive	Green olive, No Brand, Seoul, Korea	2.5
		Dried vegetable	Characteristic aromatics associated with dried vegetable	Cooked dried daikon leaves, E‐mart, Seoul, Korea	2.5
		Solomon's seal tea	Characteristic aromatics associated with brewed solomon's seal tea	Brewed Solomon's seal tea, Dongseo, Seoul, Korea	2
		Cooked daikon	Characteristic aromatics associated with cooked daikon	Cooked daikon, Jeonju, Korea	1.5
		Bracken	Characteristic aromatics associated with cooked bracken	Cooked bracken, Jeonju, Korea	3
	Minty	Bay leaf	Characteristic aromatics associated with dried bay leaf	Dried bay leaf, Peacock, Seoul, Korea	2.5
		Mint	Characteristic aromatics associated with mint leaf	Peppermint teabag, Lipton, Glasgow, United Kingdom	3
		Perilla leaf	Characteristic aromatics associated with perilla leaf	Perilla leaf, Jeonju, Korea	2.5
Roasted	Roasted	Fried black bean	Characteristic aromatics associated with fried black bean	Fried black bean, Jeonju, Korea	2.5
		Fried pork belly	Characteristic aromatics associated with fried pork belly	Fried pork belly, E‐mart, Korea	3.5
	Burnt	Burnt	Characteristic aromatics associated with burnt wood	Burnt wood	3.5
		Cigarette	Characteristic aromatics associated with cigarettes	Cigarette soaked in warm water for 30 min	2
		Coffee	Characteristic aromatics associated with instant coffee powder	Houseblend powdered coffee, Starbucks, Seattle, WA, USA	3.5
	Nutty	Nutty	Characteristic aromatics associated with roasted nut	Brazil nut, Nuts pia, Gwangju, Korea	2
		Peanut	Characteristic aromatics associated with roasted peanut	Roasted peanut, Jeonju, Korea	2.5
Grain	Grain	Puffed rice	Characteristic aromatics associated with puffed rice	Puffed rice cookie, Ildong Foodies, Seoul, Korea	1.5
		Rice powder	Characteristic aromatics associated with rice flour	Rice flour, Soybean food, Gunsan, Korea	1.5
		Salt cracker	Characteristic aromatics associated with salted cracker	Zec cracker, Lotte food, Seoul, Korea	2.5
		Seasoned cracker	Characteristic aromatics associated with seasoned cracker	Goraebob, Orion, Seoul, Korea	2.5
		Water cracker	Characteristic aromatics associated with water cracker	Carr's table water cracker, Carr's, Carlisle, United Kingdom	1.5
		Cheese snack	Characteristic aromatics associated with cheese cracker	Conchi, Crown, Asan, Korea	2
	Dough	Mashed potato	Characteristic aromatics associated with mashed potato	Mashed potato, Dongwon, Seoul, Korea	3
		Plain bread	Characteristic aromatics associated with plain bread	Soft‐sandwich plain bread, Samlip, Seoul, Korea	1
Spicy aromatics	Spicy aromatics	Dried pepper	Characteristic aromatics associated with dried pepper	Dried pepper, E‐mart, Jeonju, Korea	2.5
		Red pepper powder	Characteristic aromatics associated with red pepper powder	Chili powder, Hansaeng, Seocheon, Korea	2.5
		Spicy sauce	Characteristic aromatics associated with spicy sauce	Spice tteokbokki Sauce, Chungjungwon, Seoul, Korea	2.5
Others		Alcohol	Characteristic aromatics associated with alcohol	10% ethyl alcohol solution (v/v), Merck, Darmstadt, Germany	3.5
		Medicinal	Characteristic aromatics associated with antiseptics	Band‐aid, Johnson & Johnson, NJ, USA	1.5
		Metallic	Characteristic aromatics associated with metal	1% Ferrous sulfate solution (v/v), Merck, Darmstadt, Germany	2.5
		Phenol	Characteristic aromatics associated with phenol solution	10% phenol solution (v/v), Merck, Darmstadt, Germany	3
		Trash can	Characteristic aromatics associated with trash can	mixture of overripe vegetable extract or commercial compost‐like odor reference	3.5
		Playdough	Characteristic aromatics associated with playdough	Playdough, Hasbro, Pawtucket, RI, USA	4
		Coconut water	Characteristic aromatics associated with coconut water	Coconut water, Tipco, Thailand	2
		Soil	Characteristic aromatics associated with soil	Sterile potting soil, BFA, Goyang, Korea	2.5
	Sour	Sour aromatics	Characteristic aromatics associated with acetic acid	1% acetic acid (v/v), Merck, Darmstadt, Germany	3
		fermented milk beverage	Characteristic aromatics associated with fermented milk beverage	Yakult, HY, Seoul, Korea; intensity	2.5
		Tomato ketchup	Characteristic aromatics associated with tomato ketchup	Tomato ketchup, Ottogi, Seocheon, Korea	2.5

After the aroma lexicon, descriptor definitions, reference standards, and reference intensity values had been finalized, formal aroma intensity evaluation was conducted using Compusense Cloud software, version 2022, Compusense Inc., Ontario, Canada. Samples were presented monadically in randomized order using a Latin square design. Panelists rested for 5 min between samples and used water and unsalted crackers to minimize sensory fatigue and carryover effects. Sensory evaluation was conducted over multiple sessions, with each session lasting approximately 2 h. For each sample, the three replicate evaluations were completed on the same day, with replicates presented non‐consecutively in randomized order among other samples. All study procedures were approved by the Institutional Review Board of Jeonbuk National University (IRB #: JBNU 2020‐08‐007‐001). Written informed consent was obtained from all panelists prior to their participation in the study.

#### 2.2.3 Development of Sensory Aroma Wheel

**FIGURE 1:**
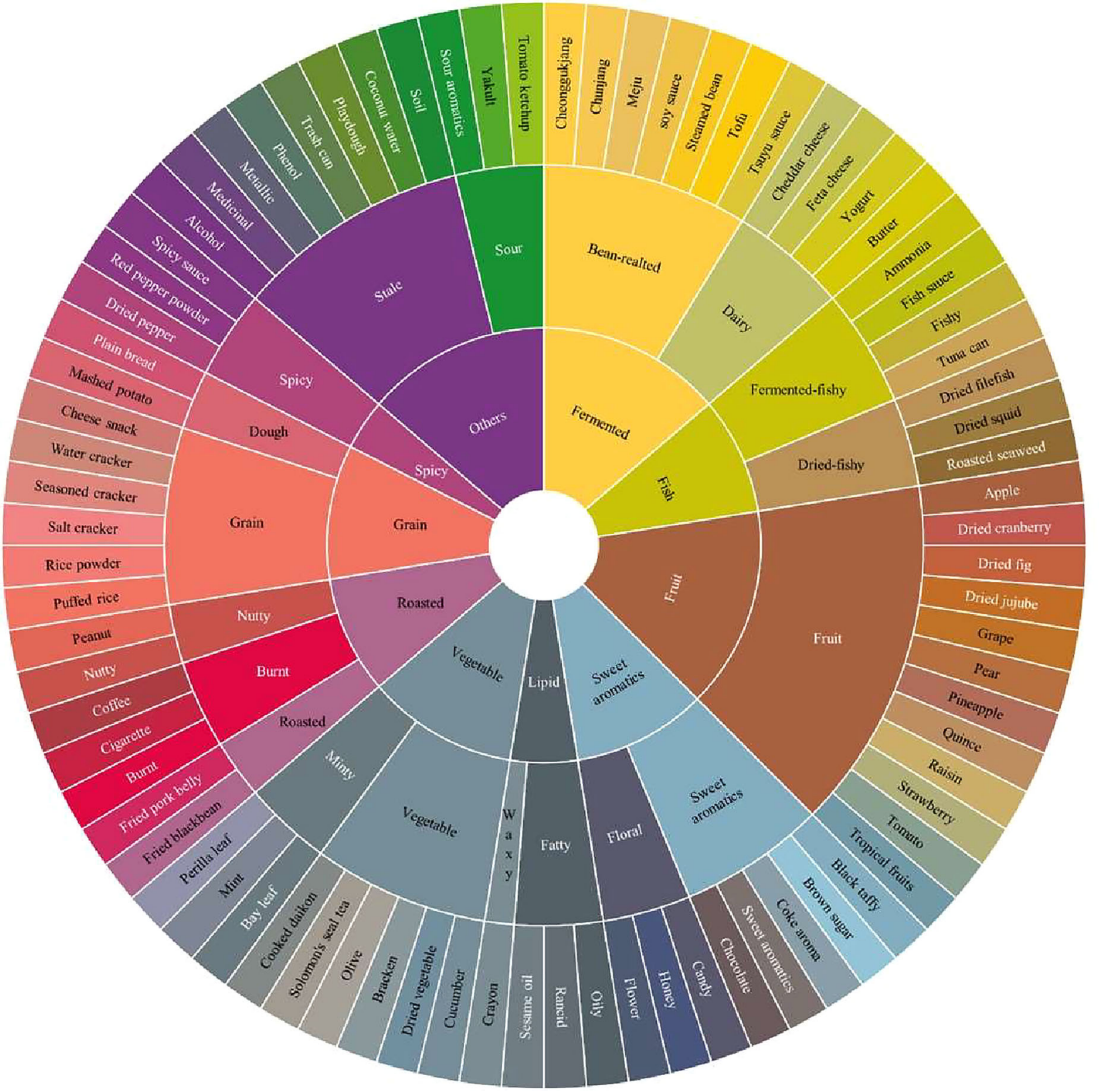
Aroma wheel of traditional *doenjang* produced in Korea.

The sensory aroma wheel was developed to organize and visualize the aroma descriptors of traditional Korean *doenjang*. The 80 aroma descriptors identified from the 81 samples were hierarchically classified according to similarities in aroma characteristics and sensory perception. The 80 aroma descriptors were grouped into higher‐order categories based on their perceptual similarity and common sensory characteristics for the development of the aroma wheel. The classification process followed previously reported sensory wheel development approaches (Koch et al. 2012). To reduce subjectivity, three sensory evaluation experts independently classified the descriptors into higher‐order sensory categories. The independent classifications were then reviewed and finalized through consensus discussion. Hierarchical cluster analysis (HCA) was also performed to explore relationships among aroma descriptors and to support the organization of higher‐order aroma categories within the sensory wheel. Descriptor clustering patterns obtained from HCA were compared with the expert‐driven classification structure. The finalized aroma wheel consisted of first‐level and second‐level sensory categories, and the resulting classification system was used to construct the sensory aroma wheel presented in Table 1 and Figure 1.

### 2.3 Applicability Assessment of the Developed Aroma Sensory Lexicon

To assess the applicability of the developed aroma sensory lexicon, 12 representative traditional *doenjang* samples were selected from the original sample set, with one or two samples chosen from each region. The panelists were not informed that the selected samples had been used previously during the lexicon development phase. The samples were selected to cover the regional distribution of the original set and to represent diverse aroma characteristics observed during lexicon development. Descriptive sensory analysis was conducted using the same panel, sample preparation, and evaluation procedures described above. The aroma descriptors identified during the applicability assessment were compared with the established lexicon to determine whether the sensory framework covered the representative aroma characteristics of traditional *doenjang*. The assessment focused on whether additional major descriptors were required outside the established lexicon and whether the identified descriptors could be assigned to the existing first‐ and second‐level aroma categories.

### 2.4 Statistical Analysis

Statistical analyses of descriptive sensory data were performed using XLSTAT software, version 2023, Lumivero, Denver, CO, USA. Panel‐level descriptor discrimination and reliability of sample mean estimates were evaluated using pooled descriptive sensory data. For each aroma descriptor, one‐way analysis of variance (ANOVA) was performed with sample as a fixed factor to determine whether the descriptor significantly discriminated among traditional *doenjang* samples. Because ANOVA was performed separately for 80 aroma descriptors, p‐values were adjusted using the Benjamini–Hochberg false discovery rate procedure to control false‐positive findings associated with multiple descriptor‐wise tests.

Effect size was estimated using eta‐squared (η^2^ = SS_sample/SS_total) and omega‐squared [ω^2^ = (SS_sample − df_sample × MS_error)/(SS_total + MS_error)] based on one‐way ANOVA results for each descriptor. Within‐sample variability was calculated from repeated observations for each sample. The reliability of sample mean estimates was approximated using an intraclass‐correlation‐based index derived from one‐way ANOVA mean squares. Because individual panelist identifiers were not retained in the final dataset, panelist‐level effects and sample‐by‐panelist interactions could not be modeled. Therefore, descriptor discrimination and reliability were evaluated at the pooled panel level using repeated observations.

Principal component analysis was conducted to visualize relationships among traditional *doenjang* samples and aroma descriptors and to identify major sensory dimensions associated with aroma quality variation. Hierarchical cluster analysis was performed using aroma intensity data to examine descriptor relationships. The distance measure and linkage method were selected according to the default settings in XLSTAT and are reported in the figure caption. The aroma sensory wheel was constructed based on the hierarchical classification of aroma descriptors developed through descriptive sensory analysis, with reference to descriptor relationships observed in HCA.

## 3 Results and Discussion

### 3.1 Development of Aroma Sensory Lexicon of Traditional *Doenjang*

A total of 80 aroma descriptors were developed through descriptive sensory analysis of 81 traditional Korean *doenjang* samples collected from different regions of Korea. The descriptors, definitions, reference standards, and reference intensity values, which were established by consensus among the trained panelists during the training sessions, are presented in Table 1. The descriptors were organized into 10 first‐level categories and 19 second‐level categories based on similarities in aroma characteristics and sensory perception.

The largest descriptor groups were fruit‐related aromas with 12 descriptors, fermented‐related aromas with 11 descriptors, and miscellaneous/sour aromas with 11 descriptors. These were followed by vegetable‐like aromas with 9 descriptors, sweet and grain‐like aromas with 8 descriptors each, fish‐related and roasted aromas with 7 descriptors each, lipid‐related aromas with 4 descriptors, and spicy aromas with 3 descriptors. This distribution shows that traditional *doenjang* aroma is not limited to fermented soybean‐related notes but includes fruity, dairy‐associated, fishy, roasted, grain‐like, vegetable‐like, sour, and other fermentation‐related characteristics.

Fermented‐related aromas formed a central sensory group and included bean‐related descriptors, such as *cheonggukjang*, *chunjang*, *meju*, soy sauce, steamed bean, tofu, and *tsuyu* sauce, and dairy‐associated descriptors, such as Cheddar cheese, Feta cheese, yogurt, and butter. These descriptors are consistent with previous studies reporting fermented soybean, cheese‐like, and buttery notes in *doenjang* and related fermented soybean products (Jang et al. 2021; Jo and Kim 2020; Kim et al. 2010; Kim et al. 2018; Lee et al. 2019; Shin et al. 2022; Shin et al. 2023). The dairy‐associated characteristics may be partly related to microbial metabolism during spontaneous fermentation, including amino acid degradation and formation of sulfur‐containing volatile compounds by lactic acid bacteria and other microorganisms (Baek 2017; Chun et al. 2020; Han et al. 2021; Hong et al. 2012; Jo et al. 2011; Jung et al. 2016; Smid and Kleerebezem 2014).

Fruit‐related descriptors, including apple, dried cranberry, dried fig, grape, pear, pineapple, raisin, strawberry, and tropical fruits, also represented a major part of the lexicon. Fruity notes have been reported in previous *doenjang* studies and may be associated with fermentation‐derived alcohols, aldehydes, esters, and lipid degradation products (Devanthi and Gkatzionis 2019; Jang et al. 2021; Jo et al. 2023; Liu 2012; Shin et al. 2022; Shin et al. 2023). Sweet, roasted, and nutty descriptors may also reflect Maillard reaction products and fermentation‐derived metabolites formed during long‐term aging (Kumazawa et al. 2013; Song et al. 2021). Overall, the developed lexicon reflects the multidimensional aroma quality of traditional *doenjang* produced through spontaneous fermentation and variable manufacturing practices.

### 3.2 Descriptor Discrimination and Reliability of Sample Mean Estimates

The developed aroma lexicon showed strong descriptor discrimination across the traditional *doenjang* samples (Table 2). Across 1,474 observations from 81 samples, all 80 aroma descriptors showed significant sample effects after Benjamini–Hochberg false discovery rate correction (*q* < 0.05). The median eta‐squared (η^2^) and omega‐squared (ω^2^) values were 0.885 and 0.878, respectively, indicating that sample differences accounted for the majority of the explainable variance in aroma intensity across descriptors. The reliability of sample mean estimates was also high, with a median ICC (1, k) of 0.993, and the mean within‐sample standard deviation was 0.050. These results indicate that the lexicon provided high descriptor discrimination, reliable sample mean estimates, and low within‐sample variability. Although individual panelist‐level performance could not be assessed because panelist identifiers were not retained in the final dataset, the pooled analysis showed high descriptor discrimination, as indicated by the large η^2^ and ω^2^ values, high reliability of sample mean estimates (median ICC [1, k] = 0.993), and low within‐sample variability (mean within‐sample SD = 0.050), suggesting that the trained panel generated consistent aroma intensity data at the panel level. The strong sample effects are consistent with the heterogeneous nature of traditional *doenjang*, which is produced under variable raw material, fermentation, microbial, and environmental conditions (Chun et al. 2020; Han et al. 2021; Kim et al. 2016; Park et al. 2002; Song et al. 2021).

**TABLE 2:** Pooled panel‐level descriptor discrimination and reliability of sample mean estimates by first‐level aroma category.

First‐level category	No. of descriptors	No. significant descriptors after FDR correction	Median η^2^	Median ω^2^	Median ICC (1,k)	Mean within‐sample SD
Fermented	11	11	0.855	0.846	0.990	0.117
Fish	7	7	0.854	0.846	0.990	0.046
Fruit	12	12	0.935	0.931	0.996	0.040
Sweet	8	8	0.832	0.822	0.988	0.055
Lipid	4	4	0.929	0.925	0.996	0.021
Vegetable	9	9	0.936	0.932	0.996	0.013
Roasted	7	7	0.907	0.901	0.994	0.018
Grain	8	8	0.932	0.928	0.996	0.021
Spicy	3	3	0.899	0.893	0.994	0.051
Others/Sour	11	11	0.839	0.830	0.989	0.076
**Overall**	**80**	**80**	**0.885**	**0.878**	**0.993**	**0.050**

*Note*: η^2^, eta‐squared; ω^2^, omega‐squared; ICC(1,k), approximate intraclass‐correlation‐based reliability index for sample mean estimates calculated from pooled repeated observations. *P*‐values for sample effects were adjusted using the Benjamini–Hochberg false discovery rate procedure.

### 3.3 Hierarchical Organization of Aroma Descriptors and Construction of the Sensory Aroma Wheel

**FIGURE 2:**
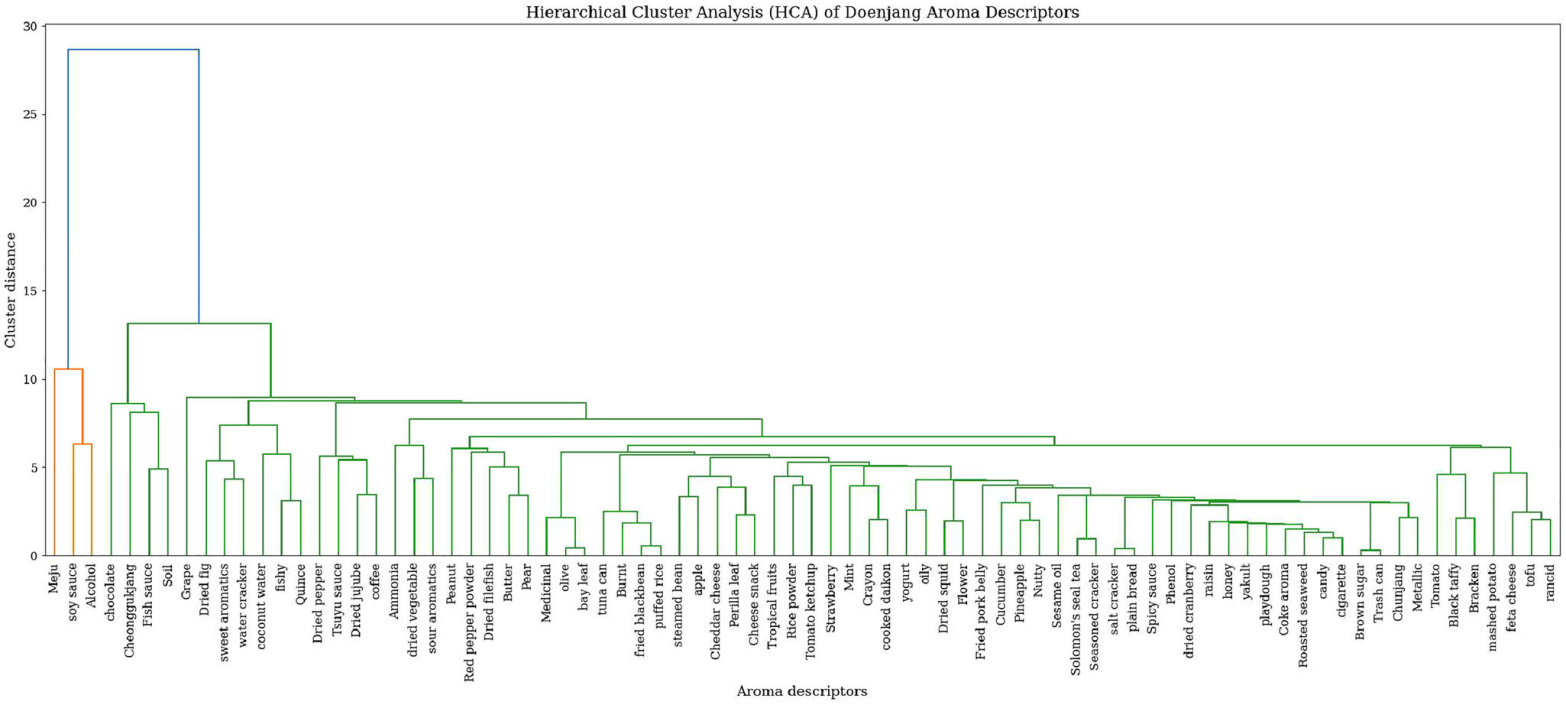
Hierarchical cluster dendrogram of aroma descriptors.

The 80 aroma descriptors were organized into a sensory aroma wheel to visualize the aroma characteristics of traditional Korean *doenjang* (Figure 1). The aroma wheel consisted of 10 first‐level categories and 19 second‐level categories. The initial classification was based on perceptual similarity, descriptor definitions, reference standards, and consensus discussion among sensory experts. Hierarchical cluster analysis (HCA) was performed to support the expert‐driven classification. The HCA dendrogram showed several descriptor clusters that corresponded well with the major sensory categories in the aroma wheel (Figure 2). Fermented soybean‐related descriptors, including *meju*, soy sauce, *cheonggukjang*, *chunjang*, steamed bean, tofu, and *tsuyu* sauce, were positioned within adjacent branches. Fruity descriptors, such as apple, grape, pear, pineapple, raisin, dried fig, and tropical fruits, also showed close clustering patterns and were largely separated from fermented soybean‐related descriptors. Dairy‐associated, fish‐related, and roasted/nutty descriptors also formed distinguishable clusters.

Comparison of HCA‐derived descriptor memberships with the sensory categories showed that the statistical clustering generally supported the perceptual organization of fermented soybean‐related, fruity, dairy‐associated, fish‐related, roasted/nutty, vegetable‐associated, and grain‐like categories (Table S2). Some descriptors were positioned across adjacent clusters, suggesting overlapping sensory characteristics. Therefore, the aroma wheel should be interpreted as a practical sensory communication tool that combines expert‐based classification and statistical descriptor relationships, rather than as a purely data‐driven structure.

Sensory wheels have been developed for complex products such as chocolate, olive oil, rooibos tea, marine oils, coffee, and pulse‐derived ingredients (Chambers et al. 2016; Chigwedere et al. 2022; De Pelsmaeker et al. 2019; Koch et al. 2012; Larssen et al. 2018; Mojet and de Jong 1994). Compared with several previous sensory wheel studies using smaller sample sets, the present study used 81 traditional *doenjang* samples, which allowed broader coverage of aroma characteristics and strengthened the practical relevance of the developed aroma wheel.

### 3.4 Multivariate Relationships Among Traditional *Doenjang* Samples and Aroma Descriptors

**FIGURE 3:**
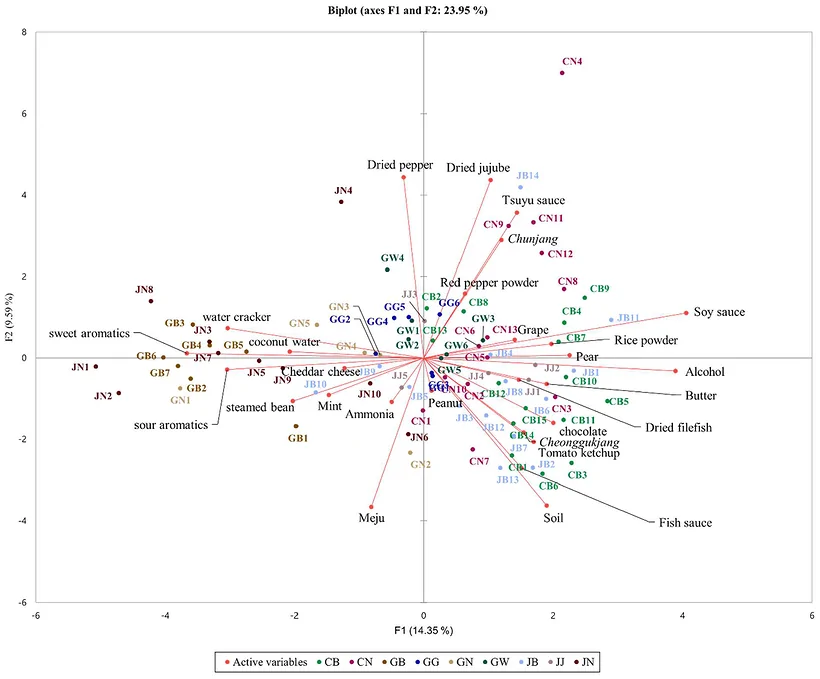
Principal component analysis biplot of major aroma lexicons with traditional *doenjang* samples produced in 9 different regions in South Korea (GG: Gyeonggi, GW: Gangwon, CN: Chungnam, CB: Chungbuk, JN: Jeonnam, JB: Jeonbuk, GN: Gyeongnam, GB: Gyeongbuk, JJ: Jeju).

Principal component analysis (PCA) was conducted to examine multivariate relationships among traditional *doenjang* samples and aroma descriptors (Figure 3). The first two principal components explained 33.54% of the total variance, with PC1 and PC2 accounting for 23.95% and 9.59%, respectively. Principal component analysis was interpreted primarily based on PC1 and PC2 because these components explained the largest proportion of variation. Although PC3 and subsequent principal components accounted for additional variation, their relatively low explained variance and more complex loading patterns limited their contribution to the overall interpretation of sample characteristics. The moderate explained variance indicates that the biplot captured only part of the total sensory variation, which is expected given the large number of descriptors and the heterogeneous sample set. The PCA biplot showed broad dispersion of the 81 samples across the sensory space, indicating substantial variation in aroma profiles among traditional *doenjang* products. Fermented soybean‐related descriptors, such as *meju*, soy sauce, *cheonggukjang*, and steamed bean, were associated with several samples and represented important aroma dimensions. Fruity descriptors, including pineapple, grape, pear, tropical fruits, and dried fruit notes, were positioned in different directions from some fermented‐related descriptors, suggesting that fruity aroma characteristics contributed to additional sample differentiation. Dairy‐associated, roasted/nutty, fish‐related, and vegetable‐associated descriptors were also distributed in different directions, indicating multidimensional aroma variation.

Although some regional tendencies were observed, the samples were not clearly separated by geographical origin, and considerable overlap occurred among regions. This suggests that traditional *doenjang* aroma profiles may be influenced not only by regional origin but also by product‐level factors such as *meju* ratio, salt concentration, aging conditions, water addition, microbial ecology, and producer‐specific fermentation practices (Han et al. 2020; Han et al. 2021; Kim et al. 2016). These results support the need for a standardized sensory framework to characterize aroma quality differences among traditional *doenjang* products.

### 3.5 Applicability Assessment of the Aroma Sensory Lexicon

**FIGURE 4:**
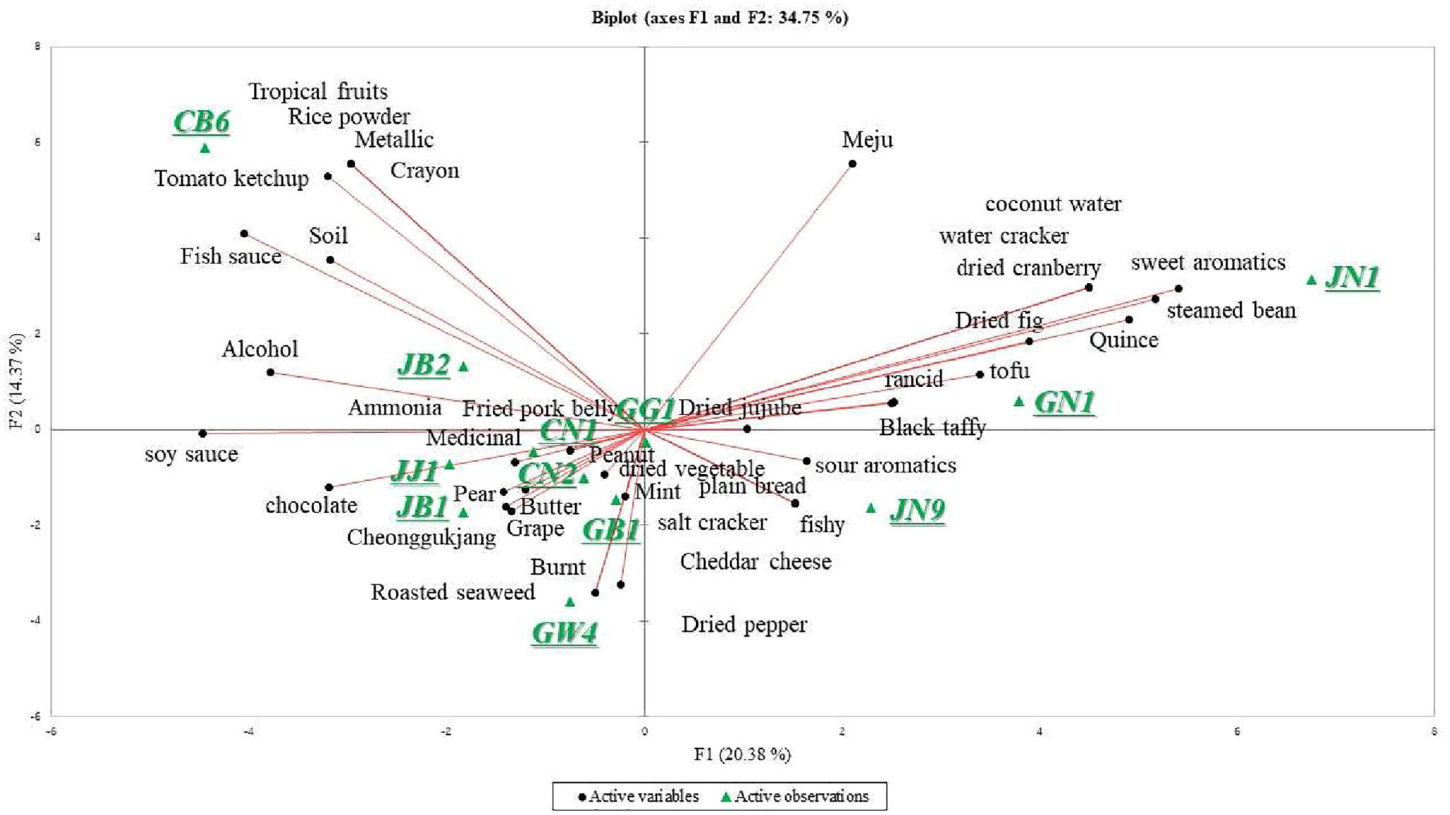
Principal component analysis biplot of the 12 representative samples used for applicability assessment. (GG: Gyeonggi, GW: Gangwon, CN: Chungnam, CB: Chungbuk, JN: Jeonnam, JB: Jeonbuk, GN: Gyeongnam, GB: Gyeongbuk, JJ: Jeju).

The applicability of the aroma sensory lexicon was assessed using 12 representative traditional *doenjang* samples selected from the original sample set, with one or two samples chosen from each region. Among the 80 descriptors in the lexicon, 40 descriptors were observed in the representative samples. No additional major aroma descriptors outside the established lexicon were generated, suggesting that the lexicon provided practical coverage of the major aroma characteristics represented by the selected samples. The PCA results for the 12 representative samples are shown in Figure 4. The first two principal components explained 49.12% of the total variance, with PC1 and PC2 accounting for 34.75% and 14.37%, respectively. Fermented soybean‐related descriptors, including *cheonggukjang*, soy sauce, and steamed bean, remained important aroma characteristics. Fruity descriptors, such as tropical fruits, grapes, and pears, were also associated with several samples, indicating that the applicability assessment captured both core fermented soybean‐related notes and additional aroma dimensions. All descriptors observed in the representative samples could be assigned to the previously established 19 second‐level sensory categories, supporting the practical coverage of the developed lexicon.

The developed lexicon and aroma wheel may support sensory communication, quality characterization, sensory training, product development, and future research on traditional fermented soybean products. Because the applicability assessment used representative samples selected from the original sample pool, further testing with independent external samples and additional trained panels would be useful to confirm broader applicability. However, this study did not directly quantify volatile compounds in the same sample set. Therefore, the proposed links between sensory descriptors and possible volatile compound classes should be interpreted as literature‐supported hypotheses. Future studies integrating this lexicon with GC–MS, GC–olfactometry, or metabolomics data would help identify the chemical drivers of the major aroma dimensions and further support quality standardization and product differentiation of traditional *doenjang*.

The aroma sensory lexicon and aroma wheel developed in this study provide a systematic framework for describing and visualizing the aroma characteristics of traditional *doenjang*. During the applicability assessment, the developed lexicon was successfully applied to representative traditional *doenjang* samples, and the identified aroma descriptors were consistently accommodated within the established aroma wheel. These findings demonstrate that the developed lexicon and aroma wheel can be effectively applied to characterize the aroma profiles of traditional *doenjang*. The applicability assessment was conducted as an internal validation using representative samples included in the lexicon development process. The primary objective was to verify that the developed lexicon and aroma wheel could be consistently applied by the trained panel and adequately describe the aroma characteristics of traditional *doenjang*. Future studies should further evaluate the robustness and generalizability of the developed lexicon through external validation using newly collected traditional *doenjang* samples and other fermented soybean products.

Although the comprehensive lexicon consisting of 80 aroma descriptors enables detailed characterization of traditional *doenjang* aroma, its routine application in industrial settings may be limited by the time and resources required for descriptive analysis. Therefore, future studies should identify a subset of representative descriptors to establish a simplified aroma lexicon that retains the essential sensory information while improving practicality for quality control and product development.

Several limitations should also be acknowledged. Individual panelist identifiers were not retained in the final analytical dataset, preventing further evaluation of panelist‐specific performance and repeatability.

Finally, commercial traditional *doenjang* samples with different production dates were stored at 4°C after purchase and evaluated within their labeled shelf life. Nevertheless, because traditional *doenjang* is a fermented product, continued biochemical and microbial changes during storage cannot be completely excluded. Future studies using samples with more tightly controlled production dates and storage conditions would further improve the consistency and applicability of the developed aroma lexicon and aroma wheel. Furthermore, the developed aroma lexicon was not directly integrated with instrumental aroma analyses, such as GC‐MS or GC‐O, using the same sample set. Consequently, the chemical basis of individual aroma descriptors could not be established in the present study. Future research combining descriptive sensory analysis with instrumental aroma analyses would provide complementary chemical evidence for the sensory descriptors and further strengthen the interpretation and practical application of the developed aroma lexicon.

## 4 Conclusion

This study developed a standardized, reference‐based aroma lexicon and sensory wheel for traditional Korean *doenjang* using 81 samples collected from different regions of Korea. The 80 aroma descriptors were organized into 10 first‐level and 19 second‐level categories, indicating the multidimensional aroma characteristics of traditional *doenjang*, including fermented soybean‐related, fruity, dairy‐associated, fish‐related, roasted, grain‐like, spicy, sour, and miscellaneous notes. HCA supported the hierarchical organization of descriptor groups, and PCA showed broad aroma variation among samples without clear separation by geographical origin alone. The applicability assessment using 12 representative samples supported the practical coverage of the lexicon without requiring additional major descriptors. The developed lexicon and aroma wheel may serve as practical tools for sensory communication, quality evaluation, product development, and future sensory–instrumental studies of traditional fermented soybean foods.

## Author Contributions

**Ji‐Sun Hwang**: formal analysis, validation, software, visualization, writing – original draft. **In‐Seo Hwang**: data curation, investigation, methodology. **Mina K. Kim**: conceptualization, funding acquisition, project administration, resources, writing – review and editing.

## Conflicts of Interest

The authors declare no conflicts of interest.

## Supporting information




**Supplementary Materials**: jfds71481‐sup‐0001‐SuppMat.docx
