# Supplementary material for: A Standardized Aroma Lexicon and Sensory Wheel for Characterizing Aroma Quality of Traditional Korean Doenjang
Source: J Food Sci. 2026 Sep 13;91(9):e71481. doi: 10.1111/1750-3841.71481 (PMC13572779; doi:10.1111/1750-3841.71481)
Supplement: Supplementary file 1 [file JFDS-91-0-s001.docx]

**Supplementary Table 1. Sample information used in the study**

| **No.** | **Sample** | **Region** | **City** | **Ingredients** |
| --- | --- | --- | --- | --- |
| 1 | GG1 | Gyeonggi  (GG) | Gimpo | *Meju* 90%, salt 10% |
| 2 | GG2 |  | Sejong | *Meju* 88%, sea salt 12% |
| 3 | GG3 |  | Yangpyeong | Soybean 98%, sea salt 2% |
| 4 | GG4 |  | Yongin | *Meju* 90%, sea salt 10% |
| 5 | GG5 |  | Icheon | Soybean 95%, salt 5% |
| 6 | GG6 |  | Pocheon | *Meju* 90%, sea salt 10% |
| 7 | GW1 | Gangwon  (GW) | Yangyang | *Meju* 88.7%, salt 11.3% |
| 8 | GW2 |  | Yeongwol | *Meju* powder 85%, salt 15% |
| 9 | GW3 |  | Chuncheon | Soybean 90%, sea salt 10% |
| 10 | GW4 |  | Pyeongchang | *Meju* powder 90%, sea salt 10% |
| 11 | GW5 |  | Hongchen | Soybean 80%, sea salt 13%, refined water 7% |
| 12 | GW6 |  | Hongchen | Soybean 80%, salt water 20% |
| 13 | CN1 | Chungnam  (CN) | Gongju | Soybean 88%, sea salt 12%, refined water |
| 14 | CN2 |  | Gongju | Soybean *meju* 75%, sea salt 15%, refined water 10% |
| 15 | CN3 |  | Geumsan | Soybean 93%, sea salt 7% |
| 16 | CN4 |  | Geumsan | Soybean 85%, sea salt 5%, rock water 10% |
| 17 | CN5 |  | Nonsan | Soybean 80%, sea salt 20% |
| 18 | CN6 |  | Nonsan | Soybean 72%, sea salt 28% |
| 19 | CN7 |  | Buyeo | *Meju* 96%, sea salt 4% |
| 20 | CN8 |  | Buyeo | *Meju* 82%, sea salt 18% |
| 21 | CN9 |  | Seosan | Soybean 95%, sea salt 5% |
| 22 | CN10 |  | Yesan | Soybean 70%, sea salt 10%, refined water 20% |
| 23 | CN11 |  | Yesan | Soybean 53%, sea salt 11%, refined water 38%, seed malt 0.1% |
| 24 | CN12 |  | Cheongyang | Soybean 87%, sea salt 13% |
| 25 | CN13 |  | Taean | Soybean 95%, sea salt 5% |
| 26 | CB1 | Chungbuk  (CB) | Goesan | Soybean 84%, sea salt 6%, refined water 10%, red pepper seed powder |
| 27 | CB2 |  | Goesan | *Meju* powder, sea salt |
| 28 | CB3 |  | Goesan | Soybean 26%, sea salt 10%, refined water 64% |
| 29 | CB4 |  | Goesan | Soybean 60%, sea salt 10%, refined water 30% |
| 30 | CB5 |  | Goesan | Soybean 90%, sea salt 10% |
| 31 | CB6 |  | Goesan | Soybean 90%, salt water 10% |
| 32 | CB7 |  | Yeongdong | *Meju*, sea salt |
| 33 | CB8 |  | Jincheon | Soybean 95%, sea salt |
| 34 | CB9 |  | Jincheon | Soybean 80%, korean soy sauce 20% |
| 35 | CB10 |  | Jincheon | Soybean 87%, sword bean 8%, salt, ethyl alcohol |
| 36 | CB11 |  | Jincheon | Soybean 80%, sea salt 7%, refined water 13% |
| 37 | CB12 |  | Jincheon | Soybean, sea salt, rock water |
| 38 | CB13 |  | Cheongju | *Meju* 70.4%, refined water 20%, salt 9.6% |
| 39 | CB14 |  | Cheongju | Soybean 85%, salt 15% |
| 40 | CB15 |  | Cheongju | *Meju* 90.5%, salt 9.5% |
| 41 | JN1 | Jeonnam  (JN) | Gokseong | Soybean 90%, sea salt 10% |
| 42 | JN2 |  | Damyang | Soybean 90%, sea salt 10% |
| 43 | JN3 |  | Suncheon | Soybean 90%, sea salt 10% |
| 44 | JN4 |  | Yeongam | Soybean 96%, sea salt 4% |
| 45 | JN5 |  | Jangheung | *Meju* 90%, sea salt 10% |
| 46 | JN6 |  | Jangheung | Fermented soybean 96%, sea salt 4% |
| 47 | JN7 |  | Haenam | *Meju* 98%, sea salt 2% |
| 48 | JN8 |  | Haenam | Soybean 98%, sea salt 2% |
| 49 | JN9 |  | Hwasun | Soybean 90%, sea salt 10% |
| 50 | JN10 |  | Hwasun | Soybean 90%, sea salt 10% |
| 51 | JB1 | Jeonbuk  (JB) | Gochang | Soybean 98%, sea salt 2% |
| 52 | JB2 |  | Gochang | *Meju*, sea salt |
| 53 | JB3 |  | Muju | Soybean 93%, sea salt 7% |
| 54 | JB4 |  | Sunchang | Soybean 70%, sea salt 15% |
| 55 | JB5 |  | Sunchang | Soybean 80%, sea salt 12%, refined water 8% |
| 56 | JB6 |  | Sunchang | *Meju* 85%, sea salt 10%, refined water 5% |
| 57 | JB7 |  | Sunchang | Soybean 65%, sea salt 18%, refined water 17% |
| 58 | JB8 |  | Sunchang | Soybean 65%, sea salt 18%, refined water 17% |
| 59 | JB9 |  | Sunchang | Soybean 78%, sea salt 12%, refined water 10% |
| 60 | JB10 |  | Sunchang | Soybean 65%, sea salt 18%, refined water 17% |
| 61 | JB11 |  | Sunchang | Soybean 80%, sea salt 10%, refined water 10% |
| 62 | JB12 |  | Sunchang | Soybean 65%, sea salt 18% |
| 63 | JB13 |  | Jeongeup | Soybean 84%, sea salt 16% |
| 64 | JB14 |  | Jeongeup | Soybean 92%, sea salt 8% |
| 65 | GN1 | Gyeongnam  (GN) | Sancheong | Soybean 88%, salt 12% |
| 66 | GN2 |  | Sancheong | Soybean 93%, sea salt 7% |
| 67 | GN3 |  | Ulsan | Soybean 95%, sea salt 5% |
| 68 | GN4 |  | Haman | *Meju* 97%, sea salt 3%, refined water |
| 69 | GN5 |  | Hamyang | Soybean 96%, bamboo salt 4% |
| 70 | GB1 | Gyeongbuk  (GB) | Gyeongju | Soybean 96%, salt 4% |
| 71 | GB2 |  | Goryeong | Soybean 98%, sea salt 2% |
| 72 | GB3 |  | Gumi | Soybean 90%, sea salt 10% |
| 73 | GB4 |  | Cheongdo | Soybean 95%, sea salt 5% |
| 74 | GB5 |  | Cheongsong | *Meju* 96%, sea salt 4% |
| 75 | GB6 |  | Cheongsong | *Meju* 96%, sea salt 4% |
| 76 | GB7 |  | Chilgok | Soybean 98%, sea salt 2% |
| 77 | JJ1 | Jeju  (JJ) | Seoguipo | *Meju* (green soybean), sea salt, refined water |
| 78 | JJ2 |  | Jeju | Soybean 90%, sea salt 10%, rock water |
| 79 | JJ3 |  | Jeju | Soybean 80%, sea salt and *meju* powder mixture 15% |
| 80 | JJ4 |  | Jeju | Soybean 87.2%, sea salt 12.8% |
| 81 | JJ5 |  | Jeju | Green soybean 88%, sea salt 12% |

**Supplementary Table S2.** Comparison between HCA-derived descriptor groupings and expert-driven aroma wheel categories

| **HCA-derived**  **descriptor grouping** | **Major descriptors included in the HCA cluster** | **Corresponding first-level aroma wheel category** | **Corresponding second-level category** | **Agreement with expert-driven aroma wheel classification** | **Interpretation** |
| --- | --- | --- | --- | --- | --- |
| Group 1 | Cheonggukjang, chunjang, meju, soy sauce, steamed bean, tofu, tsuyu sauce | Fermented | Bean-related | High | Fermented soybean-related descriptors were positioned within adjacent branches, supporting their classification as a major fermented soybean aroma dimension. |
| Group 2 | Cheddar cheese, Feta cheese, yogurt, butter | Fermented | Dairy | High | Dairy-associated descriptors showed close clustering and represented a distinct fermentation-related dairy-like aroma subdimension. |
| Group 3 | Ammonia, fish sauce, fishy, tuna can, dried filefish, dried squid, roasted seaweed | Fish | Fermented-fishy; Dried-fishy | High | Fish-related descriptors clustered according to fermented-fishy and dried-fishy characteristics, supporting their organization within the fish-related aroma category. |
| Group 4 | Apple, dried cranberry, dried fig, dried jujube, grape, pear, pineapple, quince, raisin, strawberry, tomato, tropical fruits | Fruit | Fruit | High | Fruity descriptors formed a coherent cluster separated from fermented soybean-related descriptors, indicating a distinct fruity aroma dimension in traditional doenjang. |
| Group 5 | Black taffy, brown sugar, cola aroma, sweet aromatics, chocolate, candy, honey, flower | Sweet | Sweet; Floral | Moderate to high | Sweet and floral descriptors were generally associated with sweet aromatic perception, although some descriptors may overlap with roasted or fermentation-derived aroma notes. |
| Group 6 | Oily, rancid, sesame oil, crayon | Lipid | Fatty; Waxy | Moderate | Lipid-related descriptors were grouped according to fatty, oxidized, sesame-like, and waxy aroma characteristics, but some may show overlap with roasted or miscellaneous aroma dimensions. |
| Group 7 | Cucumber, olive, dried vegetable, Solomon’s seal tea, cooked daikon, bracken, bay leaf, mint, perilla leaf | Vegetable | Vegetable; Minty | Moderate to high | Vegetable-like and herbal/minty descriptors showed statistical relationships consistent with green, cooked vegetable, and herbal aroma perception. |
| Group 8 | Fried black bean, fried pork belly, burnt, cigarette, coffee, nutty, peanut | Roasted | Roasted; Burnt; Nutty | High | Roasted, burnt, and nutty descriptors were positioned within related branches, supporting their classification as a roasted aroma dimension. |
| Group 9 | Puffed rice, rice powder, salt cracker, seasoned cracker, water cracker, cheese snack, mashed potato, plain bread | Grain | Grain; Dough | High | Grain-like and dough-like descriptors were closely associated and represented cereal, starchy, and baked product-related aroma characteristics. |
| Group 10 | Dried pepper, red pepper powder, spicy sauce | Spicy | Spicy | High | Spicy descriptors formed a small but coherent group associated with pepper- and sauce-like aroma characteristics. |
| Group 11 | Alcohol, medicinal, metallic, phenol, garbage-like/compost-like, playdough, coconut water, soil, sour aromatics, fermented milk beverage, tomato ketchup | Others/Sour | Others; Sour | Moderate | Miscellaneous and sour descriptors showed broader distribution, suggesting that these attributes captured diverse fermentation-related, acidic, earthy, medicinal, and atypical aroma characteristics not fully represented by the main categories. |

HCA-derived clusters were interpreted based on descriptor proximity in the dendrogram and compared with the expert-driven first- and second-level categories used in the aroma wheel. Agreement was classified qualitatively as high, moderate to high, or moderate according to the degree of correspondence between statistical clustering and expert-based sensory categorization.

**Supplementary Figure 1. Regional information of samples used in the study** (GG: Gyeonggi, GW: Gangwon, CN: Chungnam, CB: Chungbuk, JN: Jeonnam, JB: Jeonbuk, GN: Gyeongnam, GB: Gyeongbuk, JJ: Jeju)
